# Combination of chemotherapy and Au-nanoparticle photothermy in the visible light to tackle doxorubicin resistance in cancer cells

**DOI:** 10.1038/s41598-018-29870-0

**Published:** 2018-07-30

**Authors:** Pedro Pedrosa, Rita Mendes, Rita Cabral, Luísa M. D. R. S. Martins, Pedro V. Baptista, Alexandra R. Fernandes

**Affiliations:** 1 UCIBIO, Departamento de Ciências da Vida, Faculdade de Ciências e Tecnologia, Universidade NOVA de Lisboa, Campus de Caparica, 2829-516 Caparica, Portugal; 2 CQE, Centro de Química Estrutural, Instituto Superior Técnico, Universidade de Lisboa, Av Rovisco Pais, 1049-001 Lisboa, Portugal; 3 Área Departamental de Engenharia Química, Instituto Superior de Engenharia de Lisboa, R. Conselheiro Emídio Navarro, 1959-007 Lisboa, Portugal

## Abstract

Despite great advances in the fight against cancer, traditional chemotherapy has been hindered by the dose dependent adverse side effects that reduce the usable doses for effective therapy. This has been associated to drug resistance in tumor cells that often cause relapse and therapy failure. These drawbacks have been tackled by combining different therapeutic regiments that prevent drug resistance while decreasing the chemotherapy dose required for efficacious ablation of cancer. In fact, new metallic compounds have been in a continuous development to extend the existing chemotherapy arsenal for these combined regimens. Here, we demonstrate that combination of a metallic compound (TS265), previously characterized by our group, with photothermy circumvents cells resistant to Doxorubicin (DOX). We first engendered a colorectal carcinoma cell line (HCT116) highly resistant to DOX, whose viability was diminished after administration of TS265. Cancer cell death was potentiated by challenging these cells with 14 nm spherical gold nanoparticles followed by laser irradiation at 532 nm. The combination of TS265 with photothermy lead to 65% cell death of the DOX resistant cells without impacting healthy cells. These results support the use of combined chemotherapy and photothermy in the visible spectrum as an efficient tool for drug resistant tumors.

## Introduction

Medicinal inorganic chemistry was erupted with the discovery of cisplatin, approved for clinical use by the US Food and Drug Administration (FDA) in 1978^1^. Later, less toxic metals than platinum were introduced as promising candidates for effective therapy, such as ruthenium, gold, copper, cobalt, etc^2,3^. Since metals have unique characteristics that feature redox activity, variable coordination modes and reactivity toward organic substrates, they allow for the design of new metal-based drugs for cancer treatment^2^. Particularly, our group has focused on cobalt-based drug, not only because they exhibit a different mechanism of action than that of platinum compounds, but also because of their selective toxicity towards cancer cells^4–7^. The Co(II) coordination compound CoCl(H2O)(phendione)2][BF4] (phendione = 1,10-phenanthroline-5,6-dione) - named TS265 – showed a pro-apoptotic activity due to the induction of reactive oxygen species and DNA strand breaks with selective *in vitro* and *in vivo* anti-tumor potential towards colorectal cancer^4–6^. The use of gold nanoparticles (AuNPs) functionalized with an anti- epidermal growth factor receptor antibody potentiated the effect of this compound *in vivo* due to the selective active targeting to cancer cells^4^.

Although conventional chemotherapeutics, such as Doxorubicin (DOX), have an efficient therapeutic response, there have been increasing reports of therapy relapse, leading to increase in dosage with concomitant more severe adverse-effects^8,9^. The non-response in cancer cells has been associated to drug resistance (DR) over time. The DR mechanisms can be disease-specific to the chemotherapeutic agent or unspecific, for example, by over expressing of efflux pumps^9,10^. To tackle this issue, combined therapy using different chemotherapeutics targeting distinct cell control mechanism and/or combination of chemical and physical approaches have proven to be effective^9,11^. For instance, preclinical studies showed a synergistic interaction between heat and cytostatic treatments, therefore DNA damaging agents get their efficacy enhanced by hyperthermia, since DNA repair processes are temperature-dependent^12,13^.

Heat generation at precise tumor sites may be achieved via diverse heating sources, such as radiation (infrared, radio frequency and microwaves) and ultrasounds^14^. More precise thermal damage effect can be enhanced using strong absorption agents, such as metal nanoparticles. Gold nanoparticles, due to their Localized Surface Plasmon Resonance (LSPR), have been explored as photothermal agents^15–17^. These strategies have been mainly used in the infrared region of the spectrum due to the optical window in the near-infrared, where hemoglobin, melanin and water absorption is reduced, increasing penetration into tissues^18^. Recently, 14 nm AuNPs were used for enhanced cancer cell death via localized hyperthermia and cell damage when irradiated using a visible laser (532 nm)^12^.

**Figure 1:**
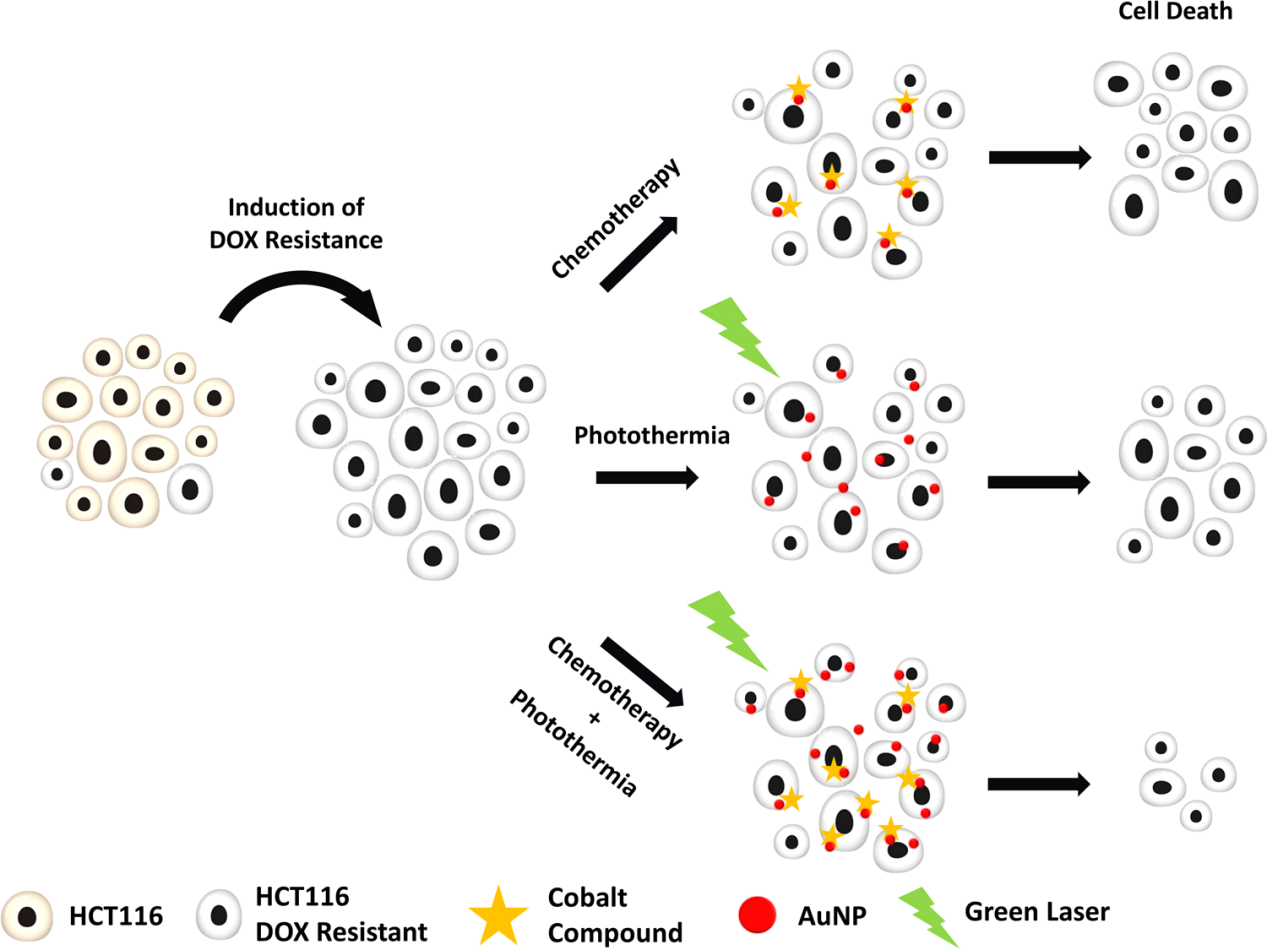
Schematics of combined chemo- hyperthermia to tackle Resistant DOX HCT116 with Cobalt compound (TS265).

In the present work, we hypothesized that the combination of a Co(II) metal compound, TS265, with anti-tumor effect and photoinduced hyperthermia using AuNPs irradiated in the visible light would result in increased tumor cell death, which could be used to circumvent DOX resistance. As such, we first established a colorectal carcinoma cell line (HCT116) highly resistant to DOX (HCT116 DOXR). Then, we used TS265 to challenge these HCT116 DOXR cells together with 14 nm spherical gold AuNPs followed by laser irradiation at 532 nm to effectively destroy DOX resistant cancer cells (Fig. 1).

## Materials and Methods

### Materials

The metal compound [Co(Phendione)_2_(H_2_O)Cl]BF_4_] (TS265) was synthesized and characterized as described in^7^. Millipore® water was used for the preparation of all aqueous solutions. Bovine Serum Albumin (BSA, 98% (w/v) with a molecular mass of 66 kDa) were purchased from Sigma-Aldrich (St Louis, MO, USA)).

### AuNPs Synthesis and Functionalization

AuNPs were synthesized, functionalized and characterized as previously described in^4,12^. Briefly, AuNPs with an average diameter of 14 nm (S.D. ±3) were functionalized with polyethylene glycol (PEG) - AuNPs@PEG - by incubating AuNPs (10 nM) with 0.028% (w/v) Sodium dodecyl sulphate (SDS), and a commercial hetero-functional PEG (SH-EG(8)-(CH_2_)_2_-COOH, Iris-Biotech, Marktredwitz, Germany). For the preparation of AuNPs@PEG@TAMRA, Tetramethylrhodamine Cadaverine, 5-(and-6)-((N-(5-Aminopentyl) Amino) Carbonyl) Tetramethylrhodamine (TAMRA, Invitrogen, Carlsbad, CA, USA) was conjugated to AuNPs via a carbodiimide (EDC)/N-hydroxysuccinimide (NHS) coupling reaction. Briefly, 20 nM of AuNPs@PEG, 1.25 mg/mL of N-hydroxysulfosuccinimide (sulfo-NHS) and 312 μg/mL of EDC were incubated in 10 mM 2-(N-morpholino)ethanesulfonic acid (MES), pH 6.1, and allowed to react for 30 minutes (min) to activate the carboxylic group, to which 10^−7^ M TAMRA was added. After an overnight period, the excess was washed by centrifuging at 14000 g for 30 min at 4 °C and removing the supernatant.

Loading of TS265 onto the AuNPs@PEG was performed according to Fernandes *et al*.^4^, thus yielding NanoTS265 (AuNP@PEG@BSA@TS265). Briefly, AuNP@PEG were functionalized with Bovine Serum Albumin (BSA), 10 μg/mL (AuNP@PEG@BSA) (MW 66,120 kDa, SigmaAldrich, St Louis, MO, USA) by EDC/NHS reaction as described. The supernatant was removed, replaced by 2.5 mM pH 6 MES buffer, and tested for protein concentration using Bradford Assay (ThermoFischer Scientific, Waltham, MA, USA). Subsequently, 6 nM AuNP@PEG@BSA, were mixed separately with 50 μM of TS265 and incubated for 1 h at 4 °C to obtain NanoTS265. After this period, solutions were washed to remove excess of TS265. The amount of TS265 in the supernatants was quantified by Inductively Coupled Plasma Mass Spectrometry (ICP-MS). The nanoparticles were characterized by UV–VIS spectroscopy, transmission electron microscopy (TEM) and dynamic light scattering (DLS) – see Supplementary Information Figs S7, S8, Tables S1 and S2.

### Cell Culture

HCT116 colorectal carcinoma cell line (ATCC CCL-247) and Primary Dermal Fibroblasts (ATCC PCS-201-010) were obtained from American Type Culture Collection (ATCC, Manassas, VA, USA). Cells were maintained in DMEM medium (Dulbecco’s Modified Eagle Medium, LifeTechnologies, Carlsbad, CA, USA) supplemented with 10% (v/v) Fetal Bovine Serum (FBS), 1% (v/v) of Penicillin (100 U/mL) -Streptomycin (100 μg/mL) (LifeTechnologies, Carlsbad, CA, USA) and under an atmosphere of 5% (v/v) CO_2_ and 99% (v/v) relative humidity at 37 °C^4^. Upon growth to confluency, cells were trypsinized, stained with 0.4% Trypan Blue solution (Invitrogen, Carlsbad, CA, USA), counted using a hemocytometer and cultured into fresh medium.

### Modified DOX resistant HCT116 cell line

HCT116 Doxorubicin-Resistant (HCT116 DOXR) cells were derived from sensitive HCT116 cells by culturing in increasing concentrations of DOX over each passage, up to a final concentration of 3.6 µM (see Fig. S1). They were maintained under the same conditions as described above and supplemented with 3.6 µM of DOX.

ABCB1 expression was analyzed by Western Blotting. Cells were washed twice with phosphate buffer saline (PBS) and resuspended in lysis buffer (150 mM NaCl, 50 mM Tris (pH 8.0), 1× phosphatase inhibitor (PhosStop, Roche, Basel, Switzerland), 5 mM ethylenediaminetetraacetic acid (EDTA), 2% (v/v) tergitol (NP-40), 1× protease inhibitor (cOmplete Mini, Roche, Basel, Switzerland), 1 mM phenylmethylsulfonyl fluoride (PMSF), and 0.1% (w/v) Dithiothreitol (DTT). Cellular extracts were sonicated and centrifuged at 5000 g for 10 min. The supernatant was recovered, and protein concentration was determined using the Pierce 660 nm Protein Assay Reagent (ThermoFisher Scientific, Waltham, MA, USA) per manufacturer’s specifications. Then, 25 μg total protein extracts were separated by SDS-PAGE in a 10% (37.5:1) acrylamide-bisacrylamide gel (Merck Millipore, Darmstadt, Germany). Following overnight electrophoretic transfer onto a 0.45-μm nitrocellulose membrane (GE Healthcare, New York, IL, USA) and blocking with 5% (w/v) milk solution in Tris-buffered saline with 0.1% (v/v) Tween 20 (TBST), blots were incubated per manufacturer’s instructions for 1 h at room temperature with primary antibodies against ABCB1 (ref. sc-55510, lot#A1514, Santa Cruz, Dallas, TX, USA) and β-actin (Ref. A5441, SigmaAldrich, St Louis, MO, USA). Membranes were further washed with 1x TBST and incubated with the appropriate secondary antibody conjugated with horseradish peroxidase (Ref. 7074, Cell Signaling Technology, Danvers, MA, USA). WesternBright ECL (Advansta, Menlo Park, CA, USA) was applied to the membranes, and signal was acquired in a Gel Doc imager (Bio-Rad, Hercules, CA, USA).

ABCB1 was specifically inhibited using Tariquidar (Ref. S8028, Selleckchem, Houston, TX, USA). HCT116 DOXR were seeded at a density of 2 × 10^4^ cells/well on a 96-well plate containing DMEM supplemented medium at 37 °C, 5% (v/v) of CO_2_ and an atmosphere of 99% (v/v) humidity for 24 h. Cell viability of HCT116 DOXR was assessed, after exposure to 3.6 μM of DOX mixed with varying concentrations of Tariquidar. MTS assay (CellTiter 96® AQueous Non-Radioactive Cell Proliferation Assay, Promega) was performed 24 h after initial stimulus. In further assays 60 nM of Tariquidar was used.

### Irradiation of cells

Cells were seeded as described above and then incubated with 8.2 nM AuNPs@PEG (ε = 2.85 × 10^−8^ M^−1^ cm^−1^) for 4 h. Subsequently, the culture medium was replaced by supplemented DMEM medium without phenol red pH indicator and irradiated with a continuous (CW) 532 nm green diode-pumped solid-state laser (DPSS) (Changchun New Industries Optoelectronics Tech. Co., LTD, Changchun, China) coupled to an optical fiber with a power set to 3.44 W.cm^−2^ for 60 s. Cell membrane integrity was immediately evaluated after irradiation by trypan blue assay and cellular viability by MTS assay 24 h after irradiation. Prior to irradiation the laser source performance was characterized as previously described^12^.

### Cell Viability

#### MTS assay

Cell viability was evaluated with CellTiter 96® AQueous Non-Radioactive Cell Proliferation Assay (Promega, Madison, WI, USA), using 3-(4,5-dimethylthiazol-2-yl)-5-(3-carboxymethoxyphenyl)-2-(4-sulfophenyl)-2H-tetrazolium, inner salt (MTS) as previously described^4,12^. Briefly, after 24 h of irradiation, cell medium was substituted by fresh medium with MTS and incubated for an additional 45 min. Absorbance was measured at 490 nm from which the absorbance of a well without cells was subtracted, and the following equation (1) applied to calculate the cell viability:

1 $${Cell}\,{Viability}\,( \% )=\frac{(\mathrm{mean}\,\mathrm{Abs}{\rm{.}}\,\mathrm{of}\,\mathrm{treatment}\,\mathrm{group})}{(\mathrm{mean}\,\mathrm{Abs}{\rm{.}}\,\mathrm{of}\,\mathrm{control}\,\mathrm{group})}\times \mathrm{100}$$

#### Trypan Blue exclusion assay

Cells were incubated with 100 µL of trypan Blue 0.4% solution (Invitrogen, Carlsbad, CA, USA) for 15 min, washed twice with PBS 1X and imaged in bright field inverted microscope (Nikon TMS, Tokyo, Japan). Pictures were taken using a Digital Camera (Sony RX100 MK2, Japan).

### Confocal Fluorescence microscopy

Cells were seeded on coverslips in 24-well plates at a density of 1×10^5^ cells/well and grown for 24 h prior to incubation with 2.5 nM AuNPs@PEG@TAMRA. Following 30 min or 4 h of incubation with AuNPs, cells were fixed in 4% paraformaldehyde (PFA) for 15 min, permeabilized with 0.1% (v/v) Triton X-100 (in PBS) for 5 min and blocked with 3% (w/v) BSA (in PBS) for 30 min. Cells were incubated with Alexa Fluor 488-Phalloidin for 1 h and mounted with DAPI-containing Fluoroshield Mounting Medium (Abcam, Cambridge, MA, USA). Confocal immunofluorescence microscopy Z-stack images were taken on a Zeiss LSM 510 META confocal point-scanning microscope. Images were processed using LSM image browser (Zeiss, Oberkochen, Germany).

### Statistics

Statistical significance of all data was verified by T-test. This analysis was performed with GraphPad Prism 7.0 (GraphPad Software, Inc) and results were considered significant for p < 0.05. Data are the average of triplicated assays and the errors are calculated by the standard deviation.

## Results and Discussion

### Chemotherapeutics efficacy against DOX resistant cancer cells

To evaluate the efficacy of our combined approach against cancer cells resistant to traditional chemotherapy, we started by inducing resistance to DOX in colorectal carcinoma cell line, HCT116. As such, we passaged HCT116 cells (ATCC CCL-247) with increasing DOX concentrations up to 3.6 μM (Fig. S1). These cells started to show small morphological alterations, with increased cytoplasm volume, higher number of vesicles, and tendency to grow in small aggregates (Fig S2). This modified DOX Resistant (HCT116 DOXR) does not shows any significant reduction in cell viability up to 6 μM of DOX by MTS assay (Fig. S3). This acquired resistance is generally the result of high expression of efflux pumps that remove xenobiotics from the intracellular space in a non-specific way, constituting a first line of cell defense against chemical insult^9^. Accordingly, western blot analysis showed upregulation of P-glycoprotein 1 (ABCB1) in HCT116 DOXR when compared to the normal cell line (Fig. S4). This upregulation could be overturned by means of a specific blocker of ABCB1 - tariquidar, rendering HCT116 DOXR cells sensitive to DOX (Fig. S5). This way, it was confirmed that HCT116 DOX acquired resistance was mainly due to the up-regulation of the efflux pump.

TS265 strong antiproliferative effect against colorectal parental cancer cells, which was efficiently translated to *in vivo* models via active nanovectorization on AuNPs^4^, prompt us to assess its cytotoxicity in HCT116 DOXR cells aiming to chemically circumvent DOX resistance in these cells. As such, we determined the relative IC_50_ of TS265 in HCT116 DOXR and compared it to the normal HCT116 (DOX sensitive cells). After 24 h exposure, the relative IC_50_ of TS265 in HCT116 DOXR and HCT116 was 0.14 and 0.13 μM, respectively. These data clearly show that TS265 is highly effective (within very low micromolar range IC_50_) against a cancer cell line resistant to a first line chemotherapeutic – DOX. The effect of TS265 in presence of tariquidar was also evaluated, which showed no difference in the IC_50_, ie TS265 effect is not affected by efflux pump activity (Table 1 and Fig. S6). Together, these data show that TS265 might be used to tackle DOX resistance in colorectal carcinoma cells.

**Table 1:** Values for the relative IC_50_ of HCT116 and HCT116 DOXR after 24 h exposure to DOX, TS265 and their individual combination with tariquidar (inhibitor).

Cell Culture	DOX	TS265	DOX + Inhibitor	TS265 + Inhibitor
HCT116	0.38 ± 0.04	0.13 ± 0.01	0.38 ± 0.03	0.12 ± 0.01
HCT116 DOXR	>6	0.14 ± 0.02	0.81 ± 0.06	0.15 ± 0.04

Concentrations are expressed in μM.

### Photothermy via AuNPs

Several reports suggest that hyperthermia has an additive\synergic effect with chemotherapy in tackling cancer^12,19–22^. Also, we have recently demonstrated that a green laser irradiation could be used to locally increase temperature of cells previously incubated with AuNPs, thus inducing cell death (Supplementary Video 1 in reference^12^). As such, we decided to use TS265 in combination with hyperthermia to increase efficacy against DOX resistant cancer cells.

**Figure 2:**
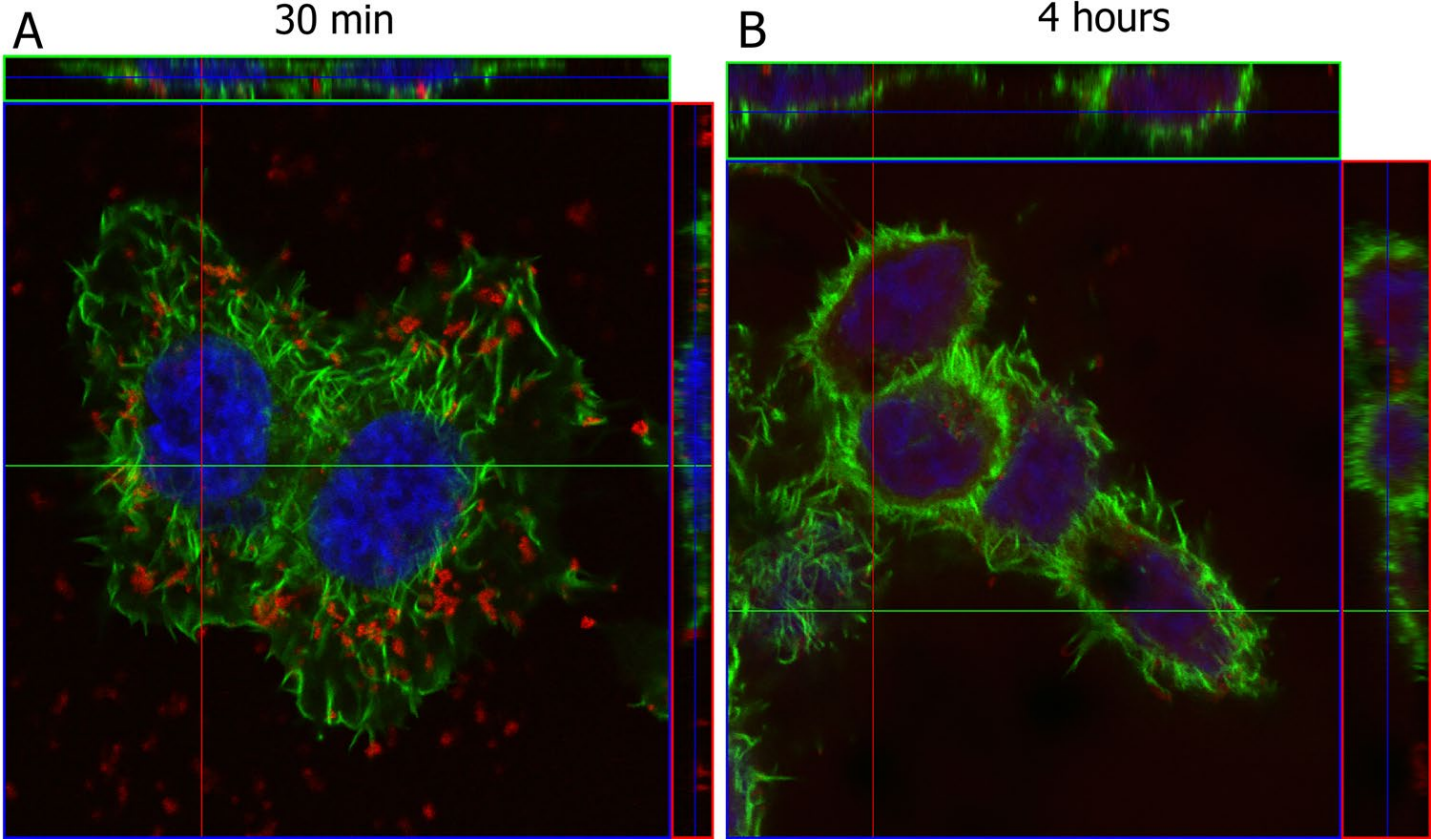
Confocal fluorescence microscopy of HCT116 cells incubated with AuNPs@PEG@TAMRA (red). Cells were stained with Alexa Fluor 488-Phalloidin (green) and the nuclear stain DAPI (blue). (**A**) Cells exposed for 30 min of AuNPs where AuNPs are mostly on cell surface and (**B**) Cells exposed to 4 h AuNPs where AuNPs are mostly inside cells.

Firstly, the time point for irradiation following cell exposure to AuNPs needed to be determined. For that, cells were incubated with AuNPs functionalized with PEG and TAMRA for tracking internalization (at 30 min and 4 h) by confocal fluorescence microscopy. Cells were further stained with Alexa Fluor 488-Phalloidin (which labels F-actin) and Z-stacks were performed. Figure 2 shows that after 30 min of incubation, the majority of nanoconjugates appear to be localized at the cell membrane. However, after 4 h of incubation, most nanoconjugates were localized closer to the nucleus (blue) and within the actin filaments (green), demonstrating internalization. Therefore, subsequent studies were performed after 4 h incubation with nanoparticles to ensure internalization of AuNPs, after which cells were washed and fresh media added to warrant removal of adherent NPs.

**Figure 3:**
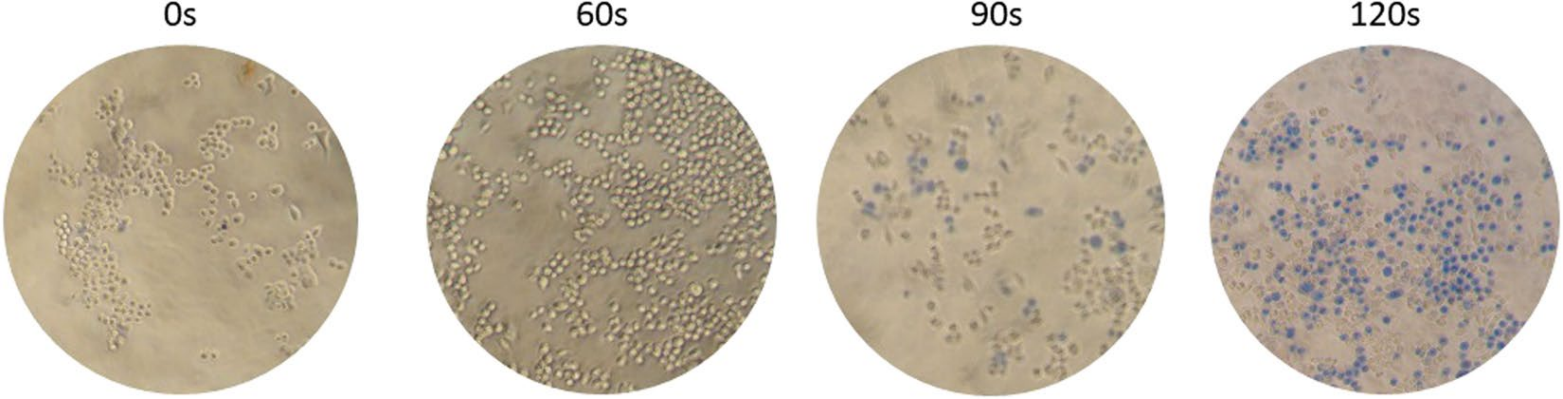
Trypan blue assay of HCT116 cells irradiated with green laser. Cells were incubated with 8.2 nM AuNPs@PEG for 4 h and medium was replaced. Later, they were irradiated with a power of 3.44 W.cm^−2^ for different exposure times and trypan blue assay was performed. For more information see Fig. S9. Cells stain with trypan blue for exposure times greater than 60 s.

After AuNPs@PEG internalization, cells were irradiated with a continuous (CW) 532 nm green diode-pumped solid-state laser (DPSS) for several exposure times after medium replacement (3.44 W.cm^−2^). Trypan blue assay (Fig. 3) showed that cells without AuNPs@PEG were not affected by irradiation for all tested exposure times, whereas cells harboring internalized AuNPs@PEG show increased membrane permeability for exposure times of more than 60 sec (s), which correlated to loss of membrane integrity, thus increased cell death (Fig. 2).

**Figure 4:**
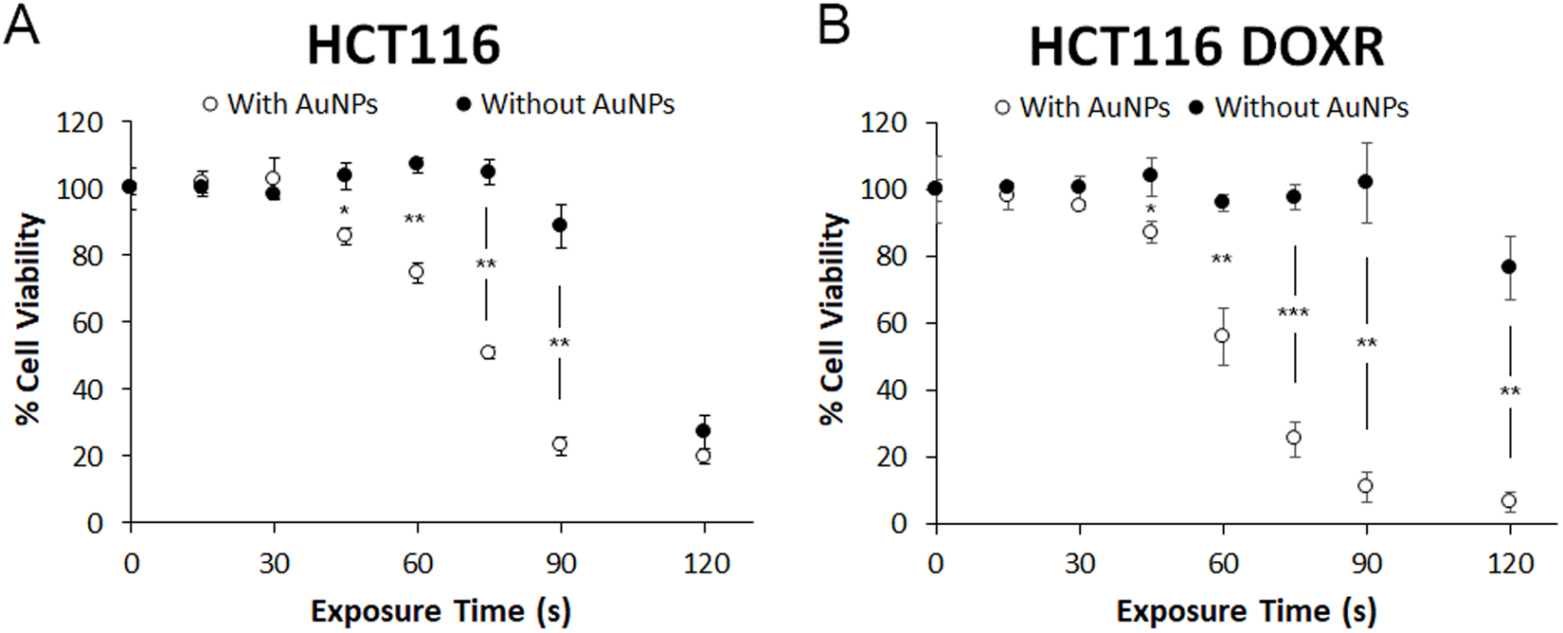
MTS assay of HCT116 cells (**A**) and HCT116 DOXR (**B**) irradiated with a power of 3.44 W.cm^−2^ with (WHITE) and without (BLACK) previous 4 h exposure to 8.2 nM of AuNPs@PEG. MTS assay was performed 24 h after irradiation. Data are the average of at least three independent assays and error bars correspondent to standard deviation.

**Figure 5:**
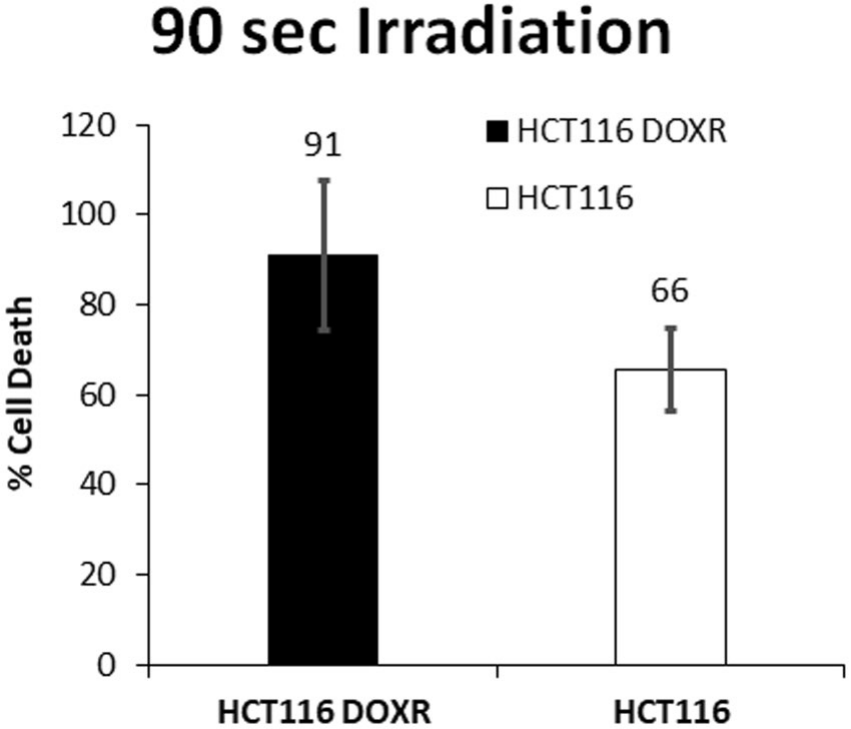
Percentage of reduction in cell viability by the MTS assay of HCT116 DOXR and HCT116 exposed to 8.2 nM of AuNPs and irradiated with 3.44 W.cm^−2^ for 90 s normalized to the same irradiation without AuNPs.

In contrast, cells without AuNPs start to show a decrease in cell viability in MTS assay, for an exposure time of 90 s. For cells with AuNPs, loss of viability starts much earlier, with 45 s of exposure (Fig. 4). The reduction in viability of cells without AuNPs might be explained due to the excitation at 532 nm of the cytochrome c^23–25^, thus compromising mitochondrial activity and not affecting membrane integrity. In the case of cells with AuNPs, loss of cell viability may be due to localized heat at the membrane, destabilizing the lipid bilayer, and permeabilizing it^26^. Also, endoskeleton damage is likely to happen, since it is where AuNPs are mostly located at 4 h^27^. It is possible to observe that HCT116 DOXR are more sensitive to AuNPs’ irradiation than HCT116, since at 90 s irradiation the difference in cell death of irradiating or not HCT116 DOXR is 91% compared with 66% for HCT116 (Fig. 5). Together these results demonstrate that irradiating ~14 nm AuNPs@PEG with a green laser promotes a photothermal injury in these cells.

### Overcoming drug resistance by chemotherapy combined to photothermy

**Figure 6:**
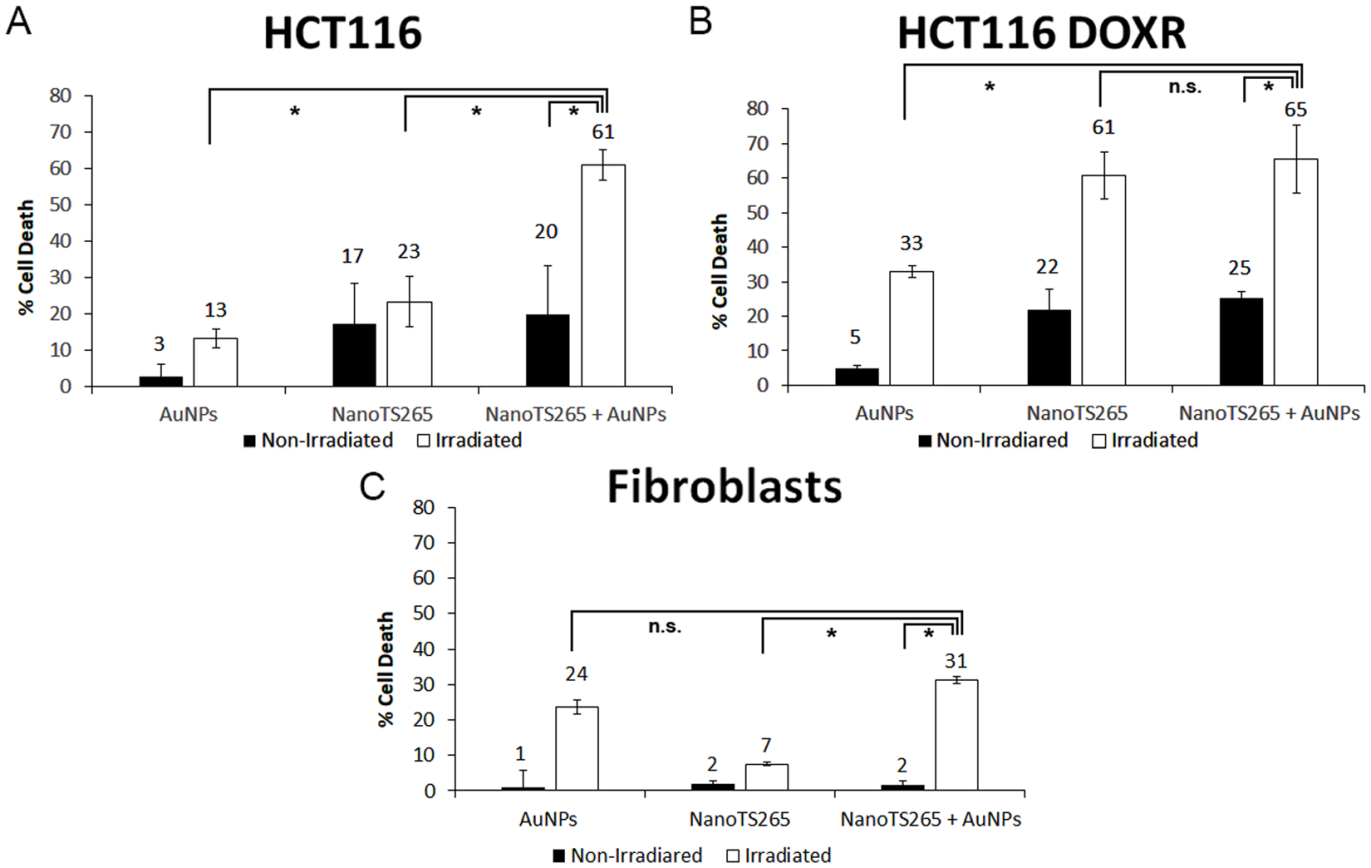
Cell death induced by the combined AuNPs based photothermy and TS265. Reduction in cell viability of (**A**) HCT116 (**B**) HCT116 DOXR and (**C**) Fibroblasts exposed to different formulations for 4 h. The concentration of AuNPs is 8.2 nM. The concentration of NanoTS265 is 0.7 nM of nanoparticles equivalent to the IC_50_ of TS265 free at 24 h. Irradiated cells were exposed to 3.44 W.cm^−2^ for 60 s (see also Fig. S10). MTS assay was performed 24 h after irradiation. Data are the average of at least three independent assays and error bars correspondent to standard deviation.

We then combined TS265 and irradiation using AuNPs to increase killing of drug resistant cancer cells without affecting normal cells. For that, we coupled TS265 with AuNPs through EDC/NHS reaction (see methods section) naming the formulation NanoTS265. We incubated HCT116, HCT116 DOXR and fibroblasts (normal cell line) with NanoTS265 (0.7 nM AuNPs) corresponding to the IC_50_ of free TS265. Data show a 20% reduction in cell viability for colorectal carcinoma cell lines – DOX sensitive and resistant, and no significant reduction in fibroblasts. This illustrates the higher cytotoxicity of this nanoformulation to cancer cells with limited toxicity to normal cells (Fig. 6).

Cells were then exposed to NanoTS265 for 4 h with subsequent irradiation for 60 s at 3.44 W.cm^−2^ with the green laser. Despite that, a temperature increase of ~5 °C was observed for all culture media, but only HCT116 DOXR suffered a significant decrease in cell viability (61% reduction), corroborating that his cell line is more sensitive to a raise in temperature.

To escalate the photothermal effect, we increased the concentration of AuNPs in the media. To warrant that, the concentration of TS265 remains constant, a mix of NanoTS265 and AuNPS@PEG was prepared at a final concentration of 8.9 nM AuNPs and added to the cell culture. Following irradiation, we attained a raise of ~12 °C for all cell lines, resulting in a reduction of cell viability of 63% for HCT116/HCT116 DOXR and 31% for fibroblasts. This increase in temperature largely increased cell death of HCT116, without significative increment to HCT116 DOXR, where irradiated NanoTS265 alone represented 61% of cell death. Together these results demonstrate the additive effect of TS265 and photothermy that allows to kill HCT116 cells and HCT116 DOXR with the same efficacy. Since fibroblasts are less sensitive to temperature variation, this approach is highly selective towards cancer cells.

## Conclusion

Drug resistance to traditional chemotherapy (e.g. DOX) is one of the major causes of anti-cancer treatment failure leading to relapse^9,28^. To overcome this, a combination of therapeutic avenues tackling different pivotal pathways in cancer development have shown promising results. Herein, we show that TS265 is a promising chemotherapeutic agent against cancer cells refractory to DOX therapy, capable of bypassing the efflux pump (ABCB1) overexpression responsible for resistance against DOX. The effect of this compound may be enhanced by combination AuNPs as photothermal agents, entering cells and promoting hyperthermia upon green (532 nm) laser irradiation. DOX resistant cancer cells are more sensitive to hyperthermia than fibroblasts. However, since we are using visible radiation, it is possible to easily target and direct irradiation to the tumor with superior precision, minimizing its effect in healthy cells, which is not possible to achieve in chemotherapy, since its effect is systemic. Combination of selective anti-cancer compound (TS265) with AuNP enhanced photothermy selectively kills resistant cancer cells while sparing healthy tissues. What is more, the additive affect allows to use smaller dose of chemotherapeutic agent, which further reduces adverse side effects associated to systemic delivery of chemotherapy. The described approach has great potential to be translate to clinical settings, since green lasers are commonly used for skin and retinal treatment with great promise to treat drug resistant tumors.

## Electronic supplementary material

Supplementary Information
