# Supplementary material for: Combination of chemotherapy and Au-nanoparticle photothermy in the visible light to tackle doxorubicin resistance in cancer cells
Source: Sci Rep. 2018 Jul 30;8:11429. doi: 10.1038/s41598-018-29870-0 (PMC6065399; doi:10.1038/s41598-018-29870-0)
Supplement: Supplementary file 1 — Supplementary Information [file 41598_2018_29870_MOESM1_ESM.docx]

# **Supporting Information**

# **Combination of chemotherapy and Au-nanoparticle photothermy in the visible light to tackle doxorubicin resistance in** **cancer cells**

Pedro Pedrosa^1^

Rita Mendes^1^

Rita Cabral^1^

Luísa M. D. R. S. Martins^2,3^

Pedro V. Baptista^1,^*

Alexandra R. Fernandes^1,^*

^1^UCIBIO, Departamento de Ciências da Vida, Faculdade de Ciências e Tecnologia, Universidade NOVA de Lisboa, Campus de Caparica, 2829-516 Caparica, Portugal; ^2^CQE, Centro de Química Estrutural, Instituto Superior Técnico, Universidade de Lisboa, Av Rovisco Pais, 1049-001 Lisboa, Portugal; ^3^Área Departamental de Engenharia Química, Instituto Superior de Engenharia de Lisboa, R. Conselheiro Emídio Navarro, 1959-007 Lisboa, Portugal.

*Corresponding authors:

Correspondence: Alexandra R Fernandes and Pedro V Baptista

UCIBIO, Departamento de Ciências da Vida, Faculdade de Ciências e Tecnologia, Universidade NOVA de Lisboa, 2829-516 Caparica, Portugal

Tel +351-21-2948530

Fax +351-21-2948530

Email ma.fernandes@fct.unl.pt**;** pmvb@fct.unl.pt

**Figure S1** **Methodology for the growth of Doxorubicin resistant HCT116**. Cells were incubated in successive passages with increasing concentrations of Doxorubicin (DOX).


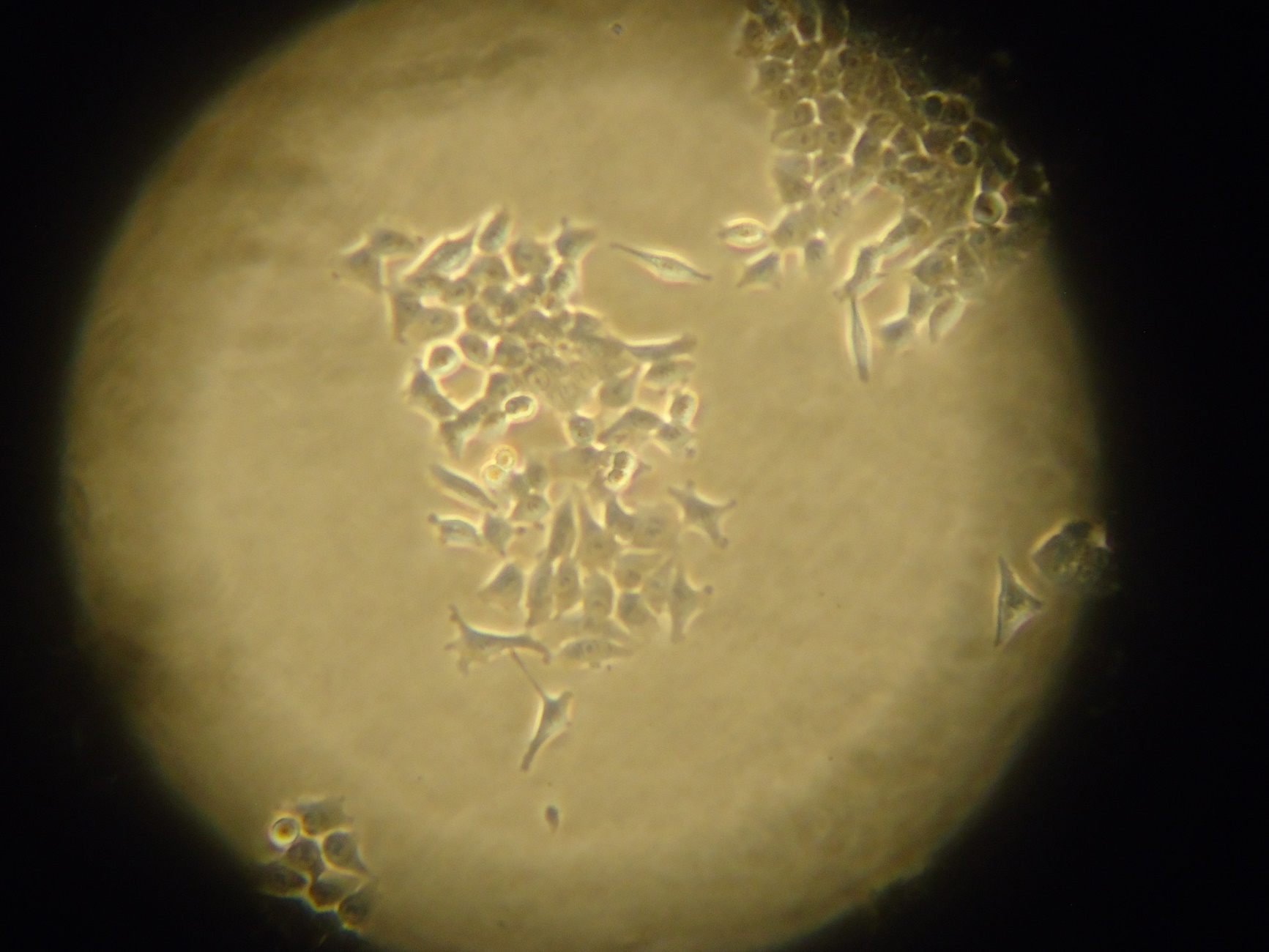


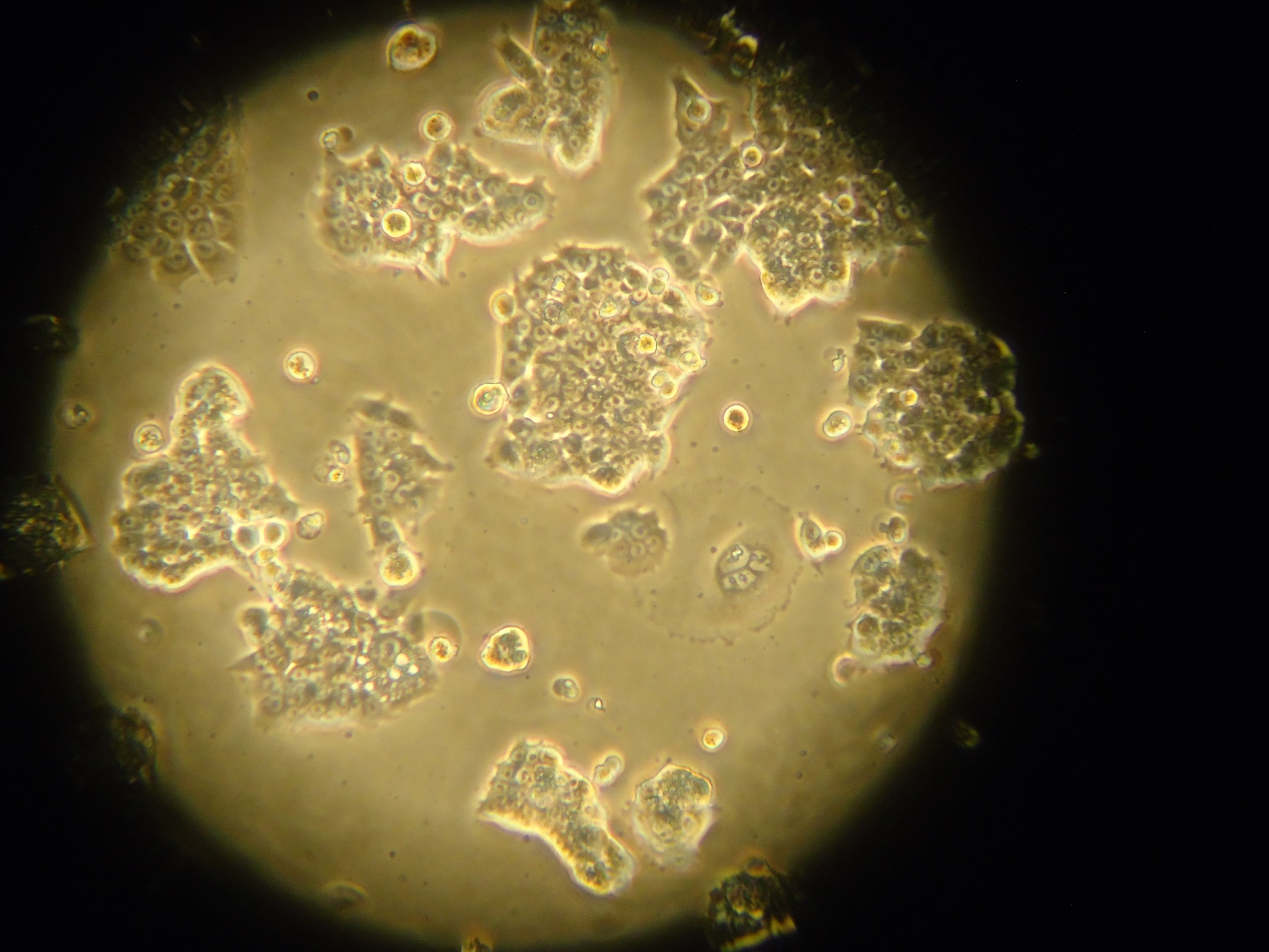

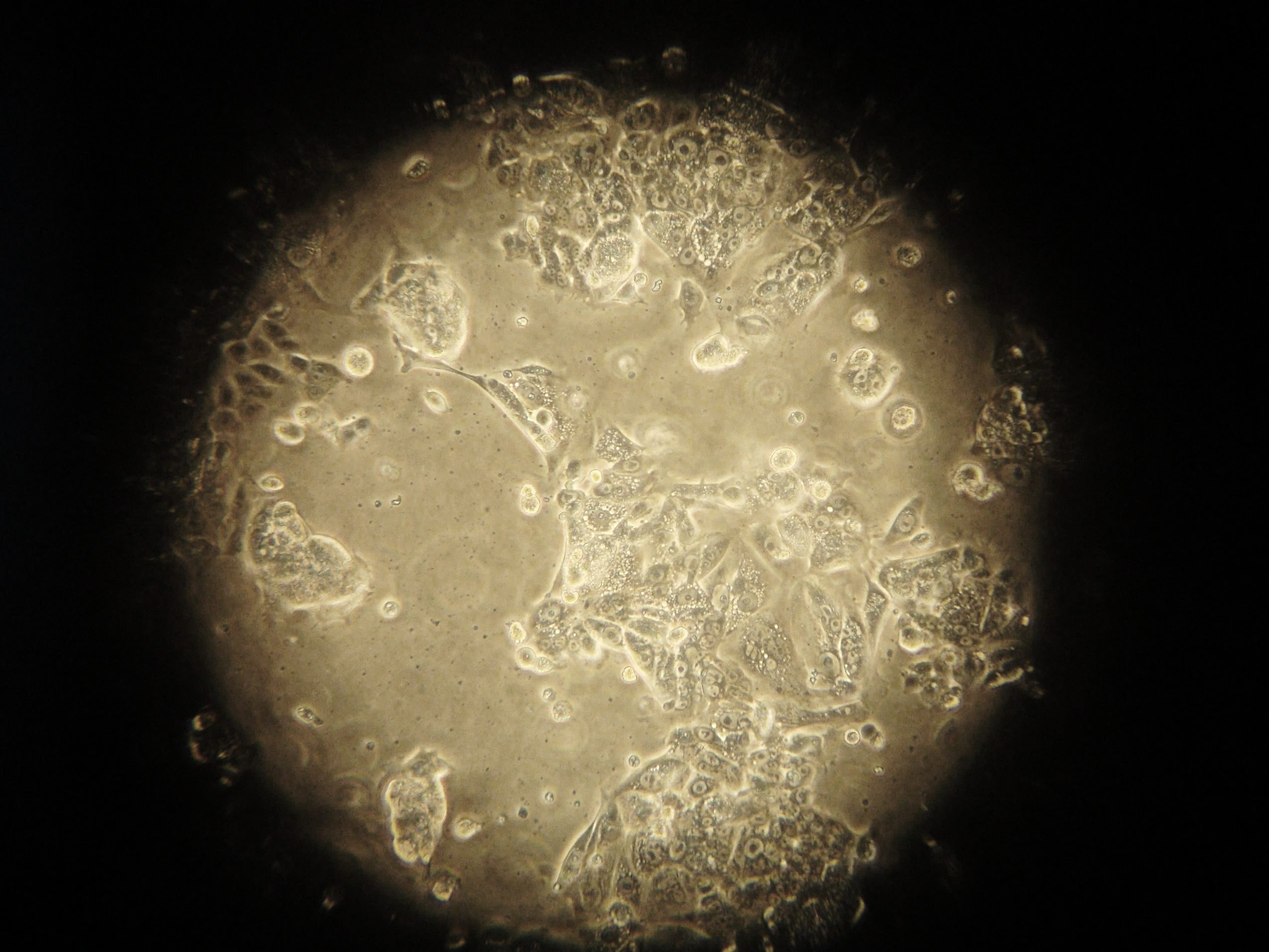


**Figure S2 Microscopy Images of HCT116 DOXR.** Microscopy images (100x) of HCT116 (top), with the characteristic star shape, and HCT116 DOXR (down-right & left) highlighting the aggregates of cells (left) and vesicles (right)

**Figure S3 MTS assay of HCT116 DOXR**. Cells were exposed to several concentrations of DOX for 24 h.

**
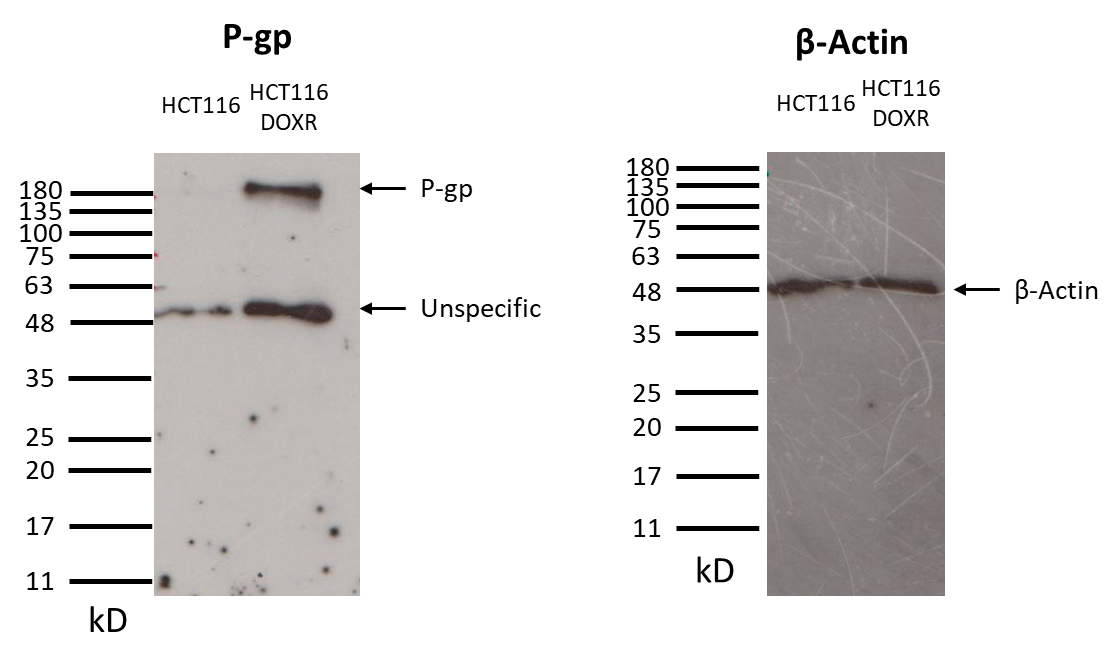
**

**Figure S4** **Western blot analysis.** Western blot of P-gp protein (ABCB1) and β-Actin (control) in HCT116 and HCT116 DOXR. Western blots images are used in compliance with the digital image and integrity policies (www.nature.com/srep/policies/index.html#digital-image). No grouping of western blots has been made. Original full-length blots are shown. No changes in contrast (exposure) were made.

**Figure S5 Cell viability of HCT116 DOXR.** Cells were exposed to 3.6 μM of DOX and varying concentrations of Tariquidar. MTS assay was performed 24 h after initial stimulus.

**Figure S6 Cell viability of HCT116 DOXR** Cells were exposed to varying concentrations of TS265 and 0 nM (WHITE) and 60 nM (BLACK) of Tariquidar. MTS assay was performed 24 h after initial stimulus.


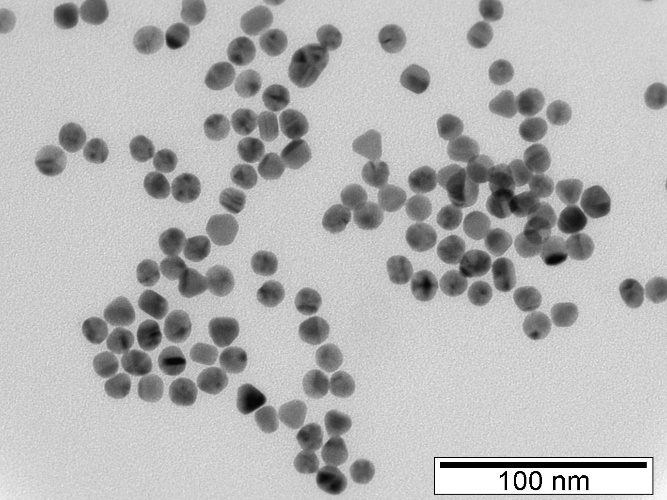


**B**

**A**

**Figure S7** **Caracterization of AuNPs by TEM** **(A)** TEM image of citrate capped AuNPs; **(B)** histogram of AuNPs distribution by diamerter (nm).

**Figure S8** **Caracterization of AuNPs by SPR peak.** Red shift in surface plasmon peak (SPR) upon each moiety addition to confirm funtionalization.

**Table S1 Characterization of AuNPs by Dynamic Light Scattering and Zeta Potential**

|  | **DLS** | | **Zeta** | |
| --- | --- | --- | --- | --- |
|  | **Average (nm)** | **St Dev** | **Average (mV)** | **St Dev** |
| AuNPs | 19.2 | 0.7 | -60.1 | 2.1 |
| AuNPs@PEG | 18.6 | 2.8 | -74.9 | 2.9 |
| AuNPs@PEG@BSA | 82.4 | 28.7 | -14.8 | 3.9 |
| NanoTS265 | 72.0 | 12.7 | -21.1 | 2.2 |
| AuNPs@PEG@TAMRA | 22.5 | 0.3 | - | - |

**Table S2 Characterization of NanoTS265.** PEG was calculated using the Ellman’s assay to measure thiols in the supernatant^1^. BSA was calculated using Pierce assay to measure protein in the supernatant. TS265 was calculated by ICP-MS of Co in the supernatant.

| **Conjugate per AuNP** | **Average** | **St Dev** |
| --- | --- | --- |
| PEG | 2320 | 280 |
| BSA | 6 | 2 |
| TS265 | 186 | 23 |


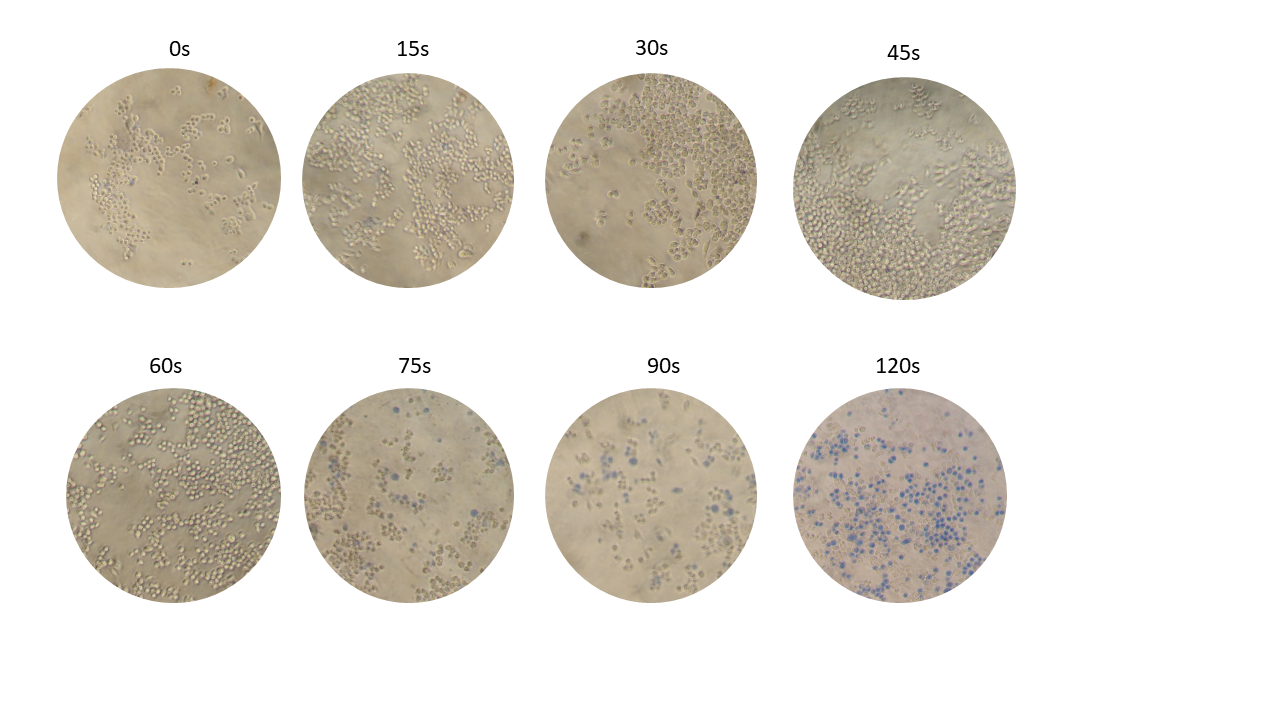


**Figure S9** **Trypan Blue assay.** HCT116 cells were incubated with 8.2 nM AuNPs@PEG for 4 h and medium was replaced. Later, they were irradiated with a power of 3.44 W.cm^-2^ for different exposure times and trypan blue assay was performed.

**A B**

**C**

**Figure S10** **Percentage of dead cells.** Reduction in cell viability of **(A)** HCT116 **(B)** HCT116 DOXR and **(C)** Fibroblasts exposed to different formulations for 4 h. The concentration of AuNPs is 0.7 nM, which is the equivalent to the AuNPs concentration of NanoTS265. The concentration of TS265 Free is the IC_50_ at 24 h. Irradiated cells were exposed to 3.44 W.cm^-2^ for 60 s. MTS assay was performed 24 h after irradiation. Data are the average of at least three independent assays and error bars correspondent to standard deviation.

Reference

1. João Conde, João Rosa, Jesús M. de la Fuente, Pedro V. Baptista. Gold-nanobeacons for simultaneous gene specific silencing and intracellular tracking of the silencing events. Biomaterials, (2013) 34(10): 2516-23 doi:10.1016/j.biomaterials.2012.12.015
